# Neuropsychological considerations in assessing and delivering non‐drug interventions for Moroccan Migrants with Dementia

**DOI:** 10.1002/alz70858_100324

**Published:** 2025-12-24

**Authors:** Mohamed Taiebine

**Affiliations:** ^1^ Euro‐Mediterranean University of Fez (UEMF), Fez, Fez, Morocco

## Abstract

**Background:**

Dementia diagnosis in Moroccan migrants presents unique challenges due to the potential influence of cultural and linguistic factors on cognitive assessment. Conventional neuropsychological assessments, often developed and validated in Western contexts, may not accurately reflect the cognitive abilities of Moroccan migrants. Factors such as limited literacy, cultural differences, and unfamiliarity with Westernized testing procedures can impact test performance, potentially leading to misdiagnosis or overdiagnosis.

**Method:**

A literature review is carried out regarding existing neuropsychological testing and care interventions for Moroccan individuals with AD. Psychometric, metrological and neuropsychological features will be detailed for neurocognitive screening and assessment tools, while a summary of care and non‐drug interventions will be described using the Rehabilitation Treatment Specification System (RTSS) framework (Taiebine et Keegan, 2024).

**Result:**

Accurate dementia diagnosis in Moroccan migrants requires the use of culturally sensitive and linguistically appropriate assessment tools. This may involve adapting existing assessments to incorporate culturally relevant stimuli and utilizing Arabic‐language or adapted Berber/Amazigh versions of tests. Effective dementia care for Moroccan migrants requires a multifaceted approach that includes culturally sensitive assessment tools, consideration of pre/post‐migration experiences and risk factors, the integration of socio‐culturally appropriate interventions and tailored neuro‐cognitive stimulation therapies.

**Conclusion:**

A comprehensive assessment should consider pre/post‐migration experiences and potential risk factors specific to Moroccan migrants, such as lower baseline education levels, social isolation, and limited access to healthcare in the host country. Addressing these challenges is crucial for improving the accuracy of dementia diagnosis and ensuring appropriate care for Moroccan migrants.

